# 2-[3,4-Dibut­oxy-5-(5-phenyl-1,3,4-oxadiazol-2-yl)-2-thien­yl]-5-phenyl-1,3,4-oxadiazole

**DOI:** 10.1107/S1600536808020254

**Published:** 2008-07-05

**Authors:** Hai-lin Li, Hai-su Zeng, Si-shun Kang, Hai-bo Wang

**Affiliations:** a College of Science, Nanjing University of Technolgy, Xinmofan Road No. 5 Nanjing, Nanjing 210009, People’s Republic of China

## Abstract

In the title compound, C_28_H_28_N_4_O_4_S, the dihedral angles between the central thio­phene ring and its pendant oxadiazole rings are 1.2 (3) and 9.8 (3)°. The dihedral angles between the oxadiazole and phenyl rings are 2.9 (3) and 1.8 (3)°. Some short intra­molecular C—H⋯O contacts occur.

## Related literature

For related literature, see: Bugatti *et al.* (2006 ▶); Brault *et al.* (2005 ▶).
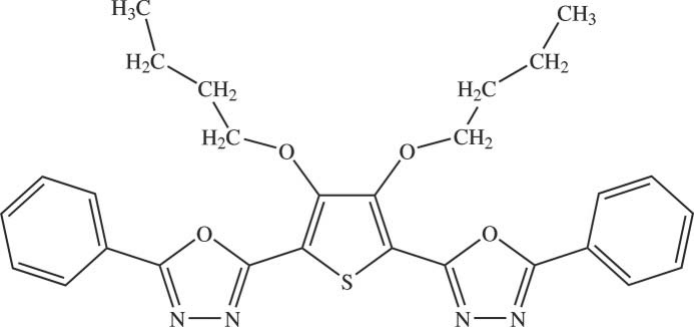


## Experimental

### 

#### Crystal data

C_28_H_28_N_4_O_4_S
*M* *_r_* = 516.60
Monoclinic, 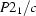

*a* = 7.6770 (15) Å
*b* = 16.871 (3) Å
*c* = 20.398 (4) Å
β = 93.77 (3)°
*V* = 2636.2 (9) Å^3^
*Z* = 4
Mo *K*α radiation
μ = 0.16 mm^−1^
*T* = 293 (2) K
0.30 × 0.10 × 0.05 mm

#### Data collection

Enraf–Nonius CAD-4 diffractometer
Absorption correction: ψ scan (North *et al.*, 1968 ▶) *T*_min_ = 0.953, *T*_max_ = 0.992
5100 measured reflections
4722 independent reflections
1918 reflections with *I* > 2σ(*I*)
*R*_int_ = 0.026
3 standard reflections every 200 reflections intensity decay: none

#### Refinement

*R*[*F* ^2^ > 2σ(*F* ^2^)] = 0.087
*wR*(*F* ^2^) = 0.203
*S* = 1.00
4722 reflections
328 parameters
216 restraints
H-atom parameters constrained
Δρ_max_ = 0.21 e Å^−3^
Δρ_min_ = −0.19 e Å^−3^

### 

Data collection: *CAD-4 Software* (Enraf–Nonius, 1989 ▶); cell refinement: *CAD-4 Software*; data reduction: *XCAD4* (Harms & Wocadlo, 1995 ▶); program(s) used to solve structure: *SHELXS97* (Sheldrick, 2008 ▶); program(s) used to refine structure: *SHELXL97* (Sheldrick, 2008 ▶); molecular graphics: *SHELXTL* (Sheldrick, 2008 ▶); software used to prepare material for publication: *SHELXL97*.

## Supplementary Material

Crystal structure: contains datablocks global, I. DOI: 10.1107/S1600536808020254/hb2756sup1.cif

Structure factors: contains datablocks I. DOI: 10.1107/S1600536808020254/hb2756Isup2.hkl

Additional supplementary materials:  crystallographic information; 3D view; checkCIF report

## Figures and Tables

**Table 1:** Hydrogen-bond geometry (Å, °)

*D*—H⋯*A*	*D*—H	H⋯*A*	*D*⋯*A*	*D*—H⋯*A*
C6—H6*A*⋯O2	0.97	2.60	2.973 (9)	103
C8—H8*B*⋯O4	0.97	2.49	3.089 (7)	120
C13—H13*A*⋯O3	0.93	2.54	2.857 (8)	100

**Fig. 1.**
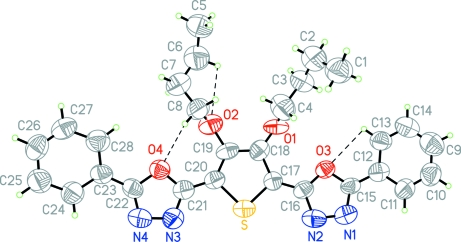
The molecular structure of (I) with displacement ellipsoids for the non-hydrogen atoms drawn at the 30% probability level. The dashed lines indicate short C—H···O contacts.

## supplementary crystallographic information

### Comment

Thiophene derivatives possess electroluminescence (Bugatti *et al.*, 2006) and biological properties (Brault *et al.*, 2005) effects. As part of our studies in this area, we report here the synthesis and crystal structure of the title compound, (I).

The molecular structure of (I) is shown in Fig. 1. The dihedral angles between the thiophene ring and its pendant O3- and O4-containing oxadiazole rings are 1.2 (3)° and 9.8 (3)°, respectively. Some short intramolecular C—H···O contacts occur (Table 1), which might help to stabilise the molecular conformation.

### Experimental

3,4-Dibutoxythiophene-2,5-dicarbohydrazide (10 mmol) was dissolved in pyridine (30 ml), and benzoyl chloride (22 mmol) was dropped into the mixture, which was heated to 348 K for 12 h. After cooling, the mixture was poured into cold water to recover a white solid.

The white solid was dissolved in phosphoryl trichloride (30 ml). The mixture was refluxed for 12 h. After cooling, the mixture was poured onto crushed ice. The crude title compound was purified by recrystalization from trichloromethane. Yield is 82% and melting point is 439 K. Yellow blocks of (I) were obtained by slow evaporation of an ethyl acetate solution.

### Refinement

All the H atoms were placed geometrically (C—H = 0.93–0.96 Å) and refined as riding with *U*_iso_(H) = 1.2*U*_eq_(C) or 1.5*U*_eq_(methyl C).

### Crystal data

**Table:**

C_28_H_28_N_4_O_4_S	*F*_000_ = 1088
*M**_r_* = 516.60	*D*_x_ = 1.302 Mg m^−^^3^
Monoclinic, *P*2_1_/*c*	Melting point: 421 K
Hall symbol: -P 2ybc	Mo *K*α radiation λ = 0.71073 Å
*a* = 7.6770 (15) Å	Cell parameters from 25 reflections
*b* = 16.871 (3) Å	θ = 8–12º
*c* = 20.398 (4) Å	µ = 0.16 mm^−^^1^
β = 93.77 (3)º	*T* = 293 (2) K
*V* = 2636.2 (9) Å^3^	Block, yellow
*Z* = 4	0.30 × 0.10 × 0.05 mm

### Data collection

**Table:**

Enraf–Nonius CAD-4 diffractometer	*R*_int_ = 0.026
Radiation source: fine-focus sealed tube	θ_max_ = 25.2º
Monochromator: graphite	θ_min_ = 1.6º
*T* = 293(2) K	*h* = −9→9
ω/2θ scans	*k* = 0→20
Absorption correction: ψ scan(North *et al.*, 1968)	*l* = 0→24
*T*_min_ = 0.953, *T*_max_ = 0.992	3 standard reflections
5100 measured reflections	every 200 reflections
4722 independent reflections	intensity decay: none
1918 reflections with *I* > 2σ(*I*)	

### Refinement

**Table:**

Refinement on *F*^2^	Secondary atom site location: difference Fourier map
Least-squares matrix: full	Hydrogen site location: inferred from neighbouring sites
*R*[*F*^2^ > 2σ(*F*^2^)] = 0.087	H-atom parameters constrained
*wR*(*F*^2^) = 0.203	*w* = 1/[σ^2^(*F*_o_^2^) + (0.05*P*)^2^ + 1.9*P*] where *P* = (*F*_o_^2^ + 2*F*_c_^2^)/3
*S* = 1.00	(Δ/σ)_max_ < 0.001
4722 reflections	Δρ_max_ = 0.21 e Å^−^^3^
328 parameters	Δρ_min_ = −0.19 e Å^−^^3^
216 restraints	Extinction correction: none
Primary atom site location: structure-invariant direct methods	

### Special details

**Table:**

Geometry. All e.s.d.'s (except the e.s.d. in the dihedral angle between two l.s. planes) are estimated using the full covariance matrix. The cell e.s.d.'s are taken into account individually in the estimation of e.s.d.'s in distances, angles and torsion angles; correlations between e.s.d.'s in cell parameters are only used when they are defined by crystal symmetry. An approximate (isotropic) treatment of cell e.s.d.'s is used for estimating e.s.d.'s involving l.s. planes.
Refinement. Refinement of *F*^2^ against ALL reflections. The weighted *R*-factor *wR* and goodness of fit *S* are based on *F*^2^, conventional *R*-factors *R* are based on *F*, with *F* set to zero for negative *F*^2^. The threshold expression of *F*^2^ > σ(*F*^2^) is used only for calculating *R*-factors(gt) *etc*. and is not relevant to the choice of reflections for refinement. *R*-factors based on *F*^2^ are statistically about twice as large as those based on *F*, and *R*- factors based on ALL data will be even larger.

### Fractional atomic coordinates and isotropic or equivalent isotropic displacement parameters (Å^2^)

**Table:**

	*x*	*y*	*z*	*U*_iso_*/*U*_eq_	
S	0.33758 (19)	−0.11570 (10)	0.45424 (7)	0.0779 (5)	
O1	0.1262 (5)	0.0185 (3)	0.57876 (19)	0.0886 (13)	
O2	0.1425 (5)	−0.1444 (3)	0.6260 (2)	0.1014 (14)	
O3	0.2220 (4)	0.1120 (2)	0.46996 (16)	0.0660 (9)	
O4	0.2964 (4)	−0.2925 (2)	0.58119 (17)	0.0745 (10)	
N1	0.3116 (6)	0.1309 (3)	0.3736 (2)	0.0812 (13)	
N2	0.3341 (6)	0.0491 (3)	0.3892 (2)	0.0889 (14)	
N3	0.3550 (7)	−0.2952 (3)	0.4784 (2)	0.0961 (16)	
N4	0.3787 (7)	−0.3714 (3)	0.5034 (3)	0.1047 (17)	
C1	0.3901 (9)	0.1927 (4)	0.6908 (3)	0.117 (2)	
H1B	0.4530	0.2145	0.7289	0.176*	
H1C	0.3584	0.2346	0.6605	0.176*	
H1D	0.4625	0.1550	0.6702	0.176*	
C2	0.2296 (10)	0.1526 (4)	0.7109 (4)	0.123 (2)	
H2A	0.2639	0.1135	0.7442	0.147*	
H2B	0.1580	0.1917	0.7313	0.147*	
C3	0.1260 (9)	0.1145 (4)	0.6610 (3)	0.103 (2)	
H3B	0.0897	0.1541	0.6284	0.124*	
H3C	0.0213	0.0955	0.6801	0.124*	
C4	0.2018 (10)	0.0482 (4)	0.6267 (4)	0.124 (3)	
H4A	0.3149	0.0655	0.6136	0.148*	
H4B	0.2234	0.0062	0.6587	0.148*	
C5	0.1109 (8)	−0.0976 (4)	0.8310 (3)	0.110 (2)	
H5A	0.0177	−0.0623	0.8406	0.165*	
H5B	0.2190	−0.0688	0.8326	0.165*	
H5C	0.1189	−0.1394	0.8629	0.165*	
C6	0.0773 (10)	−0.1303 (5)	0.7676 (4)	0.133 (3)	
H6A	0.0710	−0.0847	0.7386	0.160*	
H6B	−0.0411	−0.1503	0.7677	0.160*	
C7	0.1689 (10)	−0.1895 (4)	0.7319 (3)	0.121 (3)	
H7A	0.2515	−0.2168	0.7620	0.145*	
H7B	0.0858	−0.2282	0.7137	0.145*	
C8	0.2609 (9)	−0.1551 (4)	0.6795 (3)	0.0933 (19)	
H8A	0.3113	−0.1046	0.6933	0.112*	
H8B	0.3544	−0.1899	0.6679	0.112*	
C9	0.1390 (10)	0.4056 (5)	0.4462 (4)	0.121 (2)	
H9A	0.1199	0.4596	0.4512	0.146*	
C10	0.2049 (9)	0.3780 (4)	0.3906 (3)	0.106 (2)	
H10A	0.2278	0.4143	0.3579	0.127*	
C11	0.2395 (8)	0.3001 (4)	0.3801 (3)	0.0958 (19)	
H11A	0.2825	0.2833	0.3409	0.115*	
C12	0.2091 (6)	0.2467 (4)	0.4291 (3)	0.0761 (15)	
C13	0.1422 (8)	0.2760 (4)	0.4859 (3)	0.0948 (19)	
H13A	0.1241	0.2410	0.5200	0.114*	
C14	0.1013 (9)	0.3556 (5)	0.4938 (4)	0.112 (2)	
H14A	0.0494	0.3733	0.5310	0.134*	
C15	0.2477 (6)	0.1639 (4)	0.4222 (3)	0.0686 (14)	
C16	0.2767 (6)	0.0429 (4)	0.4483 (3)	0.0697 (14)	
C17	0.2698 (6)	−0.0266 (3)	0.4849 (3)	0.070	
C18	0.1997 (7)	−0.0394 (4)	0.5486 (3)	0.0780 (15)	
C19	0.2193 (7)	−0.1189 (4)	0.5687 (3)	0.0821 (16)	
C20	0.2853 (7)	−0.1688 (4)	0.5252 (3)	0.0726 (14)	
C21	0.3116 (7)	−0.2500 (4)	0.5263 (3)	0.0748 (15)	
C22	0.3433 (7)	−0.3675 (4)	0.5646 (3)	0.0733 (15)	
C23	0.3445 (6)	−0.4282 (4)	0.6127 (3)	0.0758 (15)	
C24	0.3920 (8)	−0.5050 (4)	0.5971 (3)	0.0933 (18)	
H24A	0.4189	−0.5153	0.5541	0.112*	
C25	0.4019 (8)	−0.5676 (4)	0.6419 (3)	0.106 (2)	
H25A	0.4390	−0.6178	0.6301	0.127*	
C26	0.3544 (8)	−0.5514 (4)	0.7040 (3)	0.099 (2)	
H26A	0.3560	−0.5921	0.7348	0.119*	
C27	0.3070 (8)	−0.4807 (5)	0.7213 (3)	0.0974 (19)	
H27A	0.2774	−0.4725	0.7642	0.117*	
C28	0.2985 (8)	−0.4146 (4)	0.6764 (3)	0.0991 (19)	
H28A	0.2635	−0.3646	0.6896	0.119*	

### Atomic displacement parameters (Å^2^)

**Table:**

	*U*^11^	*U*^22^	*U*^33^	*U*^12^	*U*^13^	*U*^23^
S	0.0735 (9)	0.0961 (11)	0.0650 (8)	0.0052 (9)	0.0125 (7)	−0.0064 (9)
O1	0.079 (3)	0.127 (4)	0.063 (2)	0.007 (3)	0.025 (2)	−0.022 (3)
O2	0.096 (3)	0.123 (4)	0.088 (3)	−0.002 (3)	0.025 (3)	0.004 (3)
O3	0.055 (2)	0.078 (2)	0.066 (2)	0.0061 (19)	0.0101 (16)	0.005 (2)
O4	0.073 (2)	0.080 (3)	0.071 (2)	0.004 (2)	0.0025 (18)	−0.003 (2)
N1	0.067 (3)	0.107 (4)	0.070 (3)	0.011 (3)	0.004 (2)	0.008 (3)
N2	0.081 (3)	0.113 (4)	0.074 (3)	0.020 (3)	0.011 (2)	−0.009 (3)
N3	0.104 (4)	0.097 (4)	0.088 (3)	0.016 (3)	0.016 (3)	0.011 (3)
N4	0.122 (4)	0.103 (4)	0.093 (3)	0.013 (3)	0.036 (3)	−0.010 (3)
C1	0.120 (6)	0.126 (6)	0.107 (5)	−0.018 (5)	0.014 (4)	−0.015 (5)
C2	0.140 (7)	0.112 (6)	0.117 (6)	−0.013 (5)	0.010 (5)	−0.011 (5)
C3	0.105 (5)	0.103 (5)	0.102 (5)	0.003 (4)	0.010 (4)	−0.020 (5)
C4	0.134 (7)	0.107 (6)	0.127 (7)	0.011 (5)	−0.019 (5)	−0.012 (5)
C5	0.096 (5)	0.122 (6)	0.112 (5)	0.004 (4)	0.008 (4)	−0.019 (5)
C6	0.145 (7)	0.129 (7)	0.128 (7)	0.001 (6)	0.028 (6)	−0.002 (6)
C7	0.149 (7)	0.128 (7)	0.087 (5)	0.006 (6)	0.010 (5)	0.010 (5)
C8	0.110 (5)	0.094 (5)	0.076 (4)	−0.012 (4)	0.007 (4)	0.003 (4)
C9	0.132 (6)	0.105 (5)	0.127 (5)	0.021 (4)	0.002 (5)	−0.011 (4)
C10	0.121 (5)	0.097 (4)	0.099 (4)	−0.008 (4)	0.003 (4)	0.013 (4)
C11	0.100 (4)	0.088 (4)	0.103 (4)	0.001 (4)	0.027 (4)	0.007 (4)
C12	0.049 (3)	0.102 (4)	0.076 (4)	−0.014 (3)	0.000 (3)	−0.008 (3)
C13	0.096 (4)	0.107 (4)	0.082 (4)	−0.015 (4)	0.014 (3)	−0.018 (4)
C14	0.112 (5)	0.118 (5)	0.105 (5)	0.005 (4)	0.010 (4)	−0.028 (4)
C15	0.056 (3)	0.087 (4)	0.063 (3)	−0.009 (3)	0.014 (3)	−0.009 (3)
C16	0.053 (3)	0.087 (4)	0.070 (3)	0.001 (3)	0.012 (3)	0.006 (3)
C17	0.070	0.070	0.070	0.000	0.005	0.000
C18	0.068 (4)	0.086 (4)	0.079 (4)	−0.015 (3)	0.001 (3)	0.005 (3)
C19	0.072 (3)	0.110 (4)	0.067 (3)	−0.004 (3)	0.026 (3)	0.002 (3)
C20	0.067 (3)	0.089 (4)	0.062 (3)	0.005 (3)	0.006 (3)	0.005 (3)
C21	0.062 (3)	0.093 (4)	0.069 (4)	0.006 (3)	0.002 (3)	0.000 (3)
C22	0.057 (3)	0.081 (4)	0.082 (4)	0.012 (3)	0.004 (3)	−0.006 (3)
C23	0.052 (3)	0.095 (4)	0.082 (4)	0.002 (3)	0.016 (3)	−0.001 (3)
C24	0.093 (4)	0.101 (4)	0.085 (4)	0.006 (4)	−0.002 (3)	−0.002 (3)
C25	0.103 (5)	0.100 (4)	0.117 (5)	−0.001 (4)	0.026 (4)	0.005 (4)
C26	0.085 (4)	0.113 (5)	0.098 (4)	−0.003 (4)	−0.005 (3)	0.021 (4)
C27	0.086 (4)	0.129 (5)	0.078 (4)	−0.011 (4)	0.012 (3)	0.010 (4)
C28	0.100 (4)	0.102 (4)	0.096 (4)	−0.003 (4)	0.018 (4)	−0.003 (4)

### Geometric parameters (Å, °)

**Table:**

S—C17	1.722 (5)	C7—C8	1.442 (7)
S—C20	1.771 (5)	C7—H7A	0.9700
O1—C4	1.212 (7)	C7—H7B	0.9700
O1—C18	1.302 (6)	C8—H8A	0.9700
O2—C8	1.385 (6)	C8—H8B	0.9700
O2—C19	1.410 (6)	C9—C14	1.331 (8)
O3—C16	1.325 (6)	C9—C10	1.355 (8)
O3—C15	1.334 (6)	C9—H9A	0.9300
O4—C21	1.341 (6)	C10—C11	1.361 (8)
O4—C22	1.365 (6)	C10—H10A	0.9300
N1—C15	1.264 (6)	C11—C12	1.376 (7)
N1—N2	1.423 (6)	C11—H11A	0.9300
N2—C16	1.315 (6)	C12—C13	1.389 (7)
N3—C21	1.300 (7)	C12—C15	1.437 (8)
N3—N4	1.390 (6)	C13—C14	1.391 (8)
N4—C22	1.296 (6)	C13—H13A	0.9300
C1—C2	1.487 (8)	C14—H14A	0.9300
C1—H1B	0.9600	C16—C17	1.392 (7)
C1—H1C	0.9600	C17—C18	1.455 (7)
C1—H1D	0.9600	C18—C19	1.408 (8)
C2—C3	1.406 (8)	C19—C20	1.346 (7)
C2—H2A	0.9700	C20—C21	1.385 (7)
C2—H2B	0.9700	C22—C23	1.418 (7)
C3—C4	1.461 (8)	C23—C28	1.387 (7)
C3—H3B	0.9700	C23—C24	1.390 (7)
C3—H3C	0.9700	C24—C25	1.395 (8)
C4—H4A	0.9700	C24—H24A	0.9300
C4—H4B	0.9700	C25—C26	1.370 (8)
C5—C6	1.414 (8)	C25—H25A	0.9300
C5—H5A	0.9600	C26—C27	1.303 (8)
C5—H5B	0.9600	C26—H26A	0.9300
C5—H5C	0.9600	C27—C28	1.442 (8)
C6—C7	1.445 (8)	C27—H27A	0.9300
C6—H6A	0.9700	C28—H28A	0.9300
C6—H6B	0.9700		
			
C17—S—C20	93.1 (3)	C10—C9—H9A	119.8
C4—O1—C18	119.5 (6)	C9—C10—C11	123.3 (7)
C8—O2—C19	113.8 (5)	C9—C10—H10A	118.3
C16—O3—C15	105.6 (4)	C11—C10—H10A	118.3
C21—O4—C22	104.5 (5)	C10—C11—C12	118.3 (7)
C15—N1—N2	107.5 (5)	C10—C11—H11A	120.8
C16—N2—N1	103.8 (5)	C12—C11—H11A	120.8
C21—N3—N4	107.5 (5)	C11—C12—C13	117.6 (6)
C22—N4—N3	106.0 (5)	C11—C12—C15	121.3 (6)
C2—C1—H1B	109.5	C13—C12—C15	121.1 (6)
C2—C1—H1C	109.5	C12—C13—C14	122.5 (7)
H1B—C1—H1C	109.5	C12—C13—H13A	118.7
C2—C1—H1D	109.5	C14—C13—H13A	118.7
H1B—C1—H1D	109.5	C9—C14—C13	117.8 (7)
H1C—C1—H1D	109.5	C9—C14—H14A	121.1
C3—C2—C1	116.7 (6)	C13—C14—H14A	121.1
C3—C2—H2A	108.1	N1—C15—O3	111.5 (5)
C1—C2—H2A	108.1	N1—C15—C12	126.8 (6)
C3—C2—H2B	108.1	O3—C15—C12	121.7 (5)
C1—C2—H2B	108.1	N2—C16—O3	111.6 (5)
H2A—C2—H2B	107.3	N2—C16—C17	125.9 (6)
C2—C3—C4	118.2 (7)	O3—C16—C17	122.5 (5)
C2—C3—H3B	107.8	C16—C17—C18	129.3 (5)
C4—C3—H3B	107.8	C16—C17—S	121.1 (4)
C2—C3—H3C	107.8	C18—C17—S	109.4 (4)
C4—C3—H3C	107.8	O1—C18—C19	128.2 (5)
H3B—C3—H3C	107.1	O1—C18—C17	120.5 (5)
O1—C4—C3	121.2 (7)	C19—C18—C17	111.3 (6)
O1—C4—H4A	107.0	C20—C19—C18	116.2 (5)
C3—C4—H4A	107.0	C20—C19—O2	123.5 (6)
O1—C4—H4B	107.0	C18—C19—O2	119.3 (5)
C3—C4—H4B	107.0	C19—C20—C21	132.0 (6)
H4A—C4—H4B	106.8	C19—C20—S	109.8 (5)
C6—C5—H5A	109.5	C21—C20—S	118.2 (5)
C6—C5—H5B	109.5	N3—C21—O4	110.7 (6)
H5A—C5—H5B	109.5	N3—C21—C20	127.8 (6)
C6—C5—H5C	109.5	O4—C21—C20	121.5 (6)
H5A—C5—H5C	109.5	N4—C22—O4	111.2 (6)
H5B—C5—H5C	109.5	N4—C22—C23	129.6 (6)
C5—C6—C7	131.6 (7)	O4—C22—C23	119.1 (5)
C5—C6—H6A	104.3	C28—C23—C24	117.1 (6)
C7—C6—H6A	104.3	C28—C23—C22	122.6 (6)
C5—C6—H6B	104.3	C24—C23—C22	120.3 (6)
C7—C6—H6B	104.3	C23—C24—C25	124.1 (6)
H6A—C6—H6B	105.6	C23—C24—H24A	118.0
C8—C7—C6	111.9 (7)	C25—C24—H24A	118.0
C8—C7—H7A	109.2	C26—C25—C24	116.7 (7)
C6—C7—H7A	109.2	C26—C25—H25A	121.7
C8—C7—H7B	109.2	C24—C25—H25A	121.7
C6—C7—H7B	109.2	C27—C26—C25	121.9 (7)
H7A—C7—H7B	107.9	C27—C26—H26A	119.1
O2—C8—C7	108.0 (6)	C25—C26—H26A	119.1
O2—C8—H8A	110.1	C26—C27—C28	122.6 (7)
C7—C8—H8A	110.1	C26—C27—H27A	118.7
O2—C8—H8B	110.1	C28—C27—H27A	118.7
C7—C8—H8B	110.1	C23—C28—C27	117.6 (6)
H8A—C8—H8B	108.4	C23—C28—H28A	121.2
C14—C9—C10	120.3 (8)	C27—C28—H28A	121.2
C14—C9—H9A	119.8		
			
C15—N1—N2—C16	0.4 (6)	C16—C17—C18—C19	−179.6 (5)
C21—N3—N4—C22	1.7 (7)	S—C17—C18—C19	4.3 (6)
C1—C2—C3—C4	63.0 (10)	O1—C18—C19—C20	173.6 (5)
C18—O1—C4—C3	178.5 (6)	C17—C18—C19—C20	−4.5 (7)
C2—C3—C4—O1	−172.1 (7)	O1—C18—C19—O2	4.8 (9)
C5—C6—C7—C8	−107.2 (9)	C17—C18—C19—O2	−173.3 (5)
C19—O2—C8—C7	−173.6 (6)	C8—O2—C19—C20	87.0 (7)
C6—C7—C8—O2	−81.9 (7)	C8—O2—C19—C18	−105.1 (6)
C14—C9—C10—C11	−1.2 (12)	C18—C19—C20—C21	−175.3 (6)
C9—C10—C11—C12	−1.3 (11)	O2—C19—C20—C21	−7.0 (10)
C10—C11—C12—C13	1.0 (9)	C18—C19—C20—S	2.5 (7)
C10—C11—C12—C15	−178.0 (6)	O2—C19—C20—S	170.8 (4)
C11—C12—C13—C14	1.9 (9)	C17—S—C20—C19	0.2 (4)
C15—C12—C13—C14	−179.1 (6)	C17—S—C20—C21	178.3 (5)
C10—C9—C14—C13	4.0 (11)	N4—N3—C21—O4	−3.1 (7)
C12—C13—C14—C9	−4.4 (10)	N4—N3—C21—C20	175.8 (5)
N2—N1—C15—O3	−0.7 (6)	C22—O4—C21—N3	3.2 (6)
N2—N1—C15—C12	177.7 (5)	C22—O4—C21—C20	−175.8 (5)
C16—O3—C15—N1	0.7 (6)	C19—C20—C21—N3	170.1 (6)
C16—O3—C15—C12	−177.7 (5)	S—C20—C21—N3	−7.5 (8)
C11—C12—C15—N1	0.4 (9)	C19—C20—C21—O4	−11.1 (10)
C13—C12—C15—N1	−178.6 (5)	S—C20—C21—O4	171.3 (4)
C11—C12—C15—O3	178.6 (5)	N3—N4—C22—O4	0.3 (7)
C13—C12—C15—O3	−0.4 (8)	N3—N4—C22—C23	179.2 (5)
N1—N2—C16—O3	0.1 (6)	C21—O4—C22—N4	−2.1 (6)
N1—N2—C16—C17	179.2 (5)	C21—O4—C22—C23	178.9 (5)
C15—O3—C16—N2	−0.4 (6)	N4—C22—C23—C28	−178.3 (6)
C15—O3—C16—C17	−179.6 (5)	O4—C22—C23—C28	0.5 (8)
N2—C16—C17—C18	−176.2 (5)	N4—C22—C23—C24	1.3 (9)
O3—C16—C17—C18	2.9 (9)	O4—C22—C23—C24	−179.9 (5)
N2—C16—C17—S	−0.5 (8)	C28—C23—C24—C25	−2.4 (9)
O3—C16—C17—S	178.5 (4)	C22—C23—C24—C25	178.0 (6)
C20—S—C17—C16	−179.0 (5)	C23—C24—C25—C26	2.7 (10)
C20—S—C17—C18	−2.6 (4)	C24—C25—C26—C27	−1.9 (10)
C4—O1—C18—C19	74.6 (9)	C25—C26—C27—C28	0.9 (11)
C4—O1—C18—C17	−107.4 (7)	C24—C23—C28—C27	1.1 (8)
C16—C17—C18—O1	2.1 (9)	C22—C23—C28—C27	−179.2 (5)
S—C17—C18—O1	−174.0 (4)	C26—C27—C28—C23	−0.5 (10)

### Hydrogen-bond geometry (Å, °)

**Table:**

*D*—H···*A*	*D*—H	H···*A*	*D*···*A*	*D*—H···*A*
C6—H6A···O2	0.97	2.60	2.973 (9)	103
C8—H8B···O4	0.97	2.49	3.089 (7)	120
C13—H13A···O3	0.93	2.54	2.857 (8)	100
